# Supplementary material for: The Impact of Immunofunctional Phenotyping on the Malfunction of the Cancer Immunity Cycle in Breast Cancer
Source: Cancers (Basel). 2020 Dec 31;13(1):110. doi: 10.3390/cancers13010110 (PMC7795596; doi:10.3390/cancers13010110)
Supplement: Supplementary file 1 [file cancers-13-00110-s001.pdf]

Supplementary Materials: The Impact of Immunofunctional Phenotyping on the Malfunction of the Cancer Immunity Cycle in Breast Cancer

Takashi Takeshita, Toshihiko Torigoe, Li Yan, Jing Li Huang, Hiroko Yamashita and Kazuaki Takabe

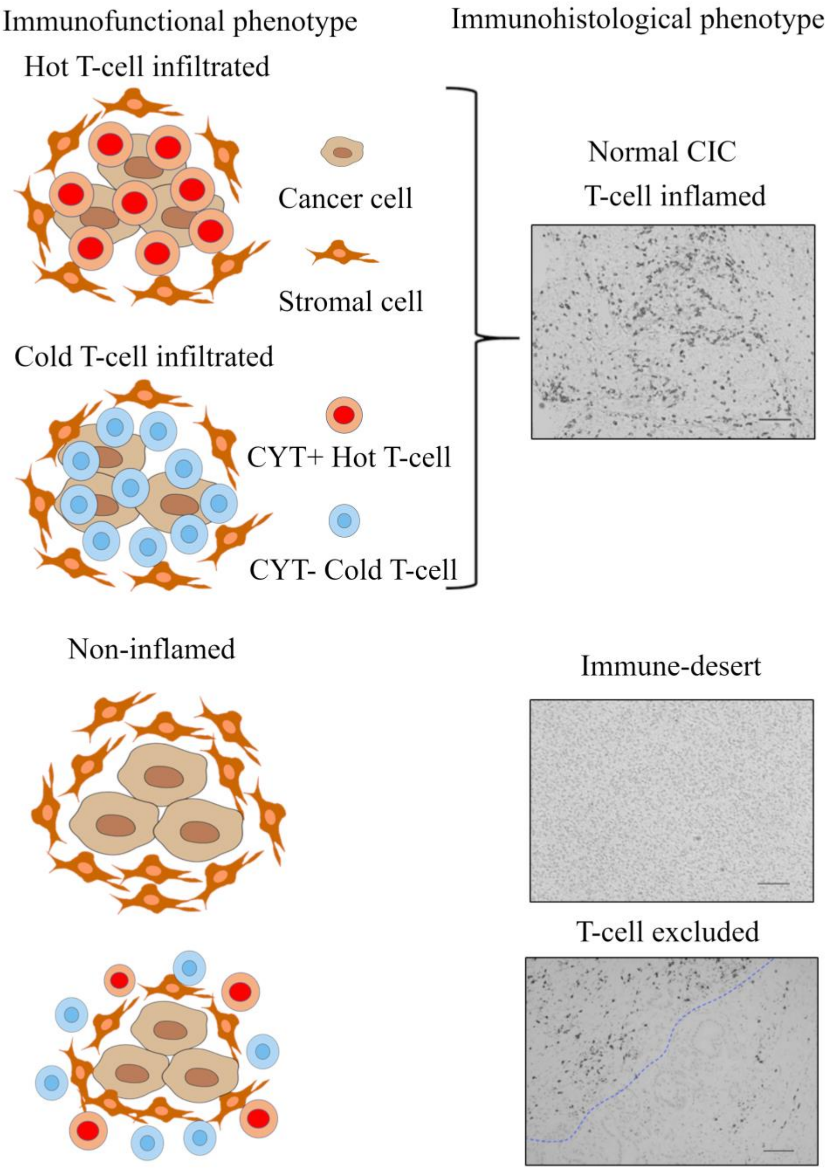

Figure S1. The definition of immunofunctional phenotype and immunohistrogical phenotype. Immunofunctional phenotype was categorized as follows: Hot T-cell infiltrated, a group of patients who had high CYT, Cold T-cell infiltrated, a group of patients who had high frequency of CD8+ T cells and low CYT, and Non-inflamed, a group of patients who had low frequency of CD8+ T cells and low CYT; left panels. Immunohistological phenotype was categorized as follows: T-cell inflamed is characterized by the presence of abundant immune cells. Immune desert is characterized by a paucity of T cells in the parenchyma or the stroma of the tumor. T-cell excluded is characterized by the presence of abundant immune cells, which do not penetrate the parenchyma of

Citation: Takeshita, T.; Torigoe, T.; Yan, L.; Huang, J.L.; Yamashita, H.; Takabe, K. Impact of Immunofunctional Phenotyping the Malfunction of the Cancer Immunity Cycle in Breast Cancer. *Cancers* **2021**, *13*, 110. https://doi.org/10.3390/cancers13010110

Received: 1 December 2020
Accepted: 28 December 2020
Published: 31 December 2020

Publisher's Note: MDPI stays neutral with regard to jurisdictional claims in published maps and institutional affiliations.

Copyright: © 2020 by the authors. Licensee MDPI, Basel, Switzerland. This article is an open access article distributed under the terms and conditions of the Creative Commons Attribution (CC BY) license (http://creativecommons.org/licenses/by/4.0/).

these tumors, but instead are retained in the stroma surrounding the nest of tumor cells. Right panels show the immunostaining of CD8+ T cells. Hot T-cell/Cold T-cell infiltrated corresponds to 'T-cell inflamed' tumors and Non-inflamed corresponds to 'Immune desert' and 'T-cell excluded' tumors in immunohistological phenotype. Scale bar represents 200 µm. Abbreviations: CYT, immune cytolytic activity, CIC, cancer immunity cycle.

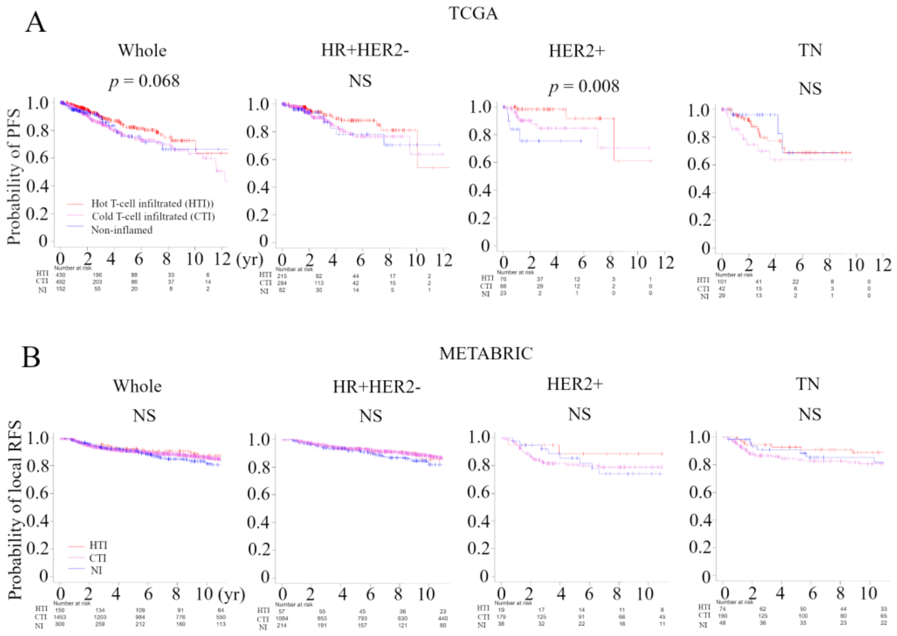

Figure S2. Analysis of the relationship between the immunofunctional phenotype and survival in TCGA and METABRIC cohorts. Kaplan-Meier plots of the association of CIC phenotype with PFS in TCGA (**A**) and local RFS in METABRIC (**B**); (left to right) the whole cohort, the HR+HER2- group, the HER2+ group, and the TN group. Immunofunctional phenotype was categorized as follows: Hot T-cell infiltrated, a group of patients who had high CYT, Cold T-cell infiltrated, a group of patients who had high frequency of CD8+ T cells and low CYT, and Non-inflamed, a group of patients who had low frequency of CD8+ T cells and low CYT. Abbreviations: CIC, cancer immunity cycle; TCGA, The Cancer Genome Atlas; METABRIC, Molecular Taxonomy of Breast Cancer International Consortium; PFS, progression free survival; BCSS, breast cancer specific survival; RFS, recurrent-free survival; HR, hormone receptor; HER2, human epidermal growth factor receptor 2; TN, triple negative; CYT, immune cytolytic activity; NS, not significant.

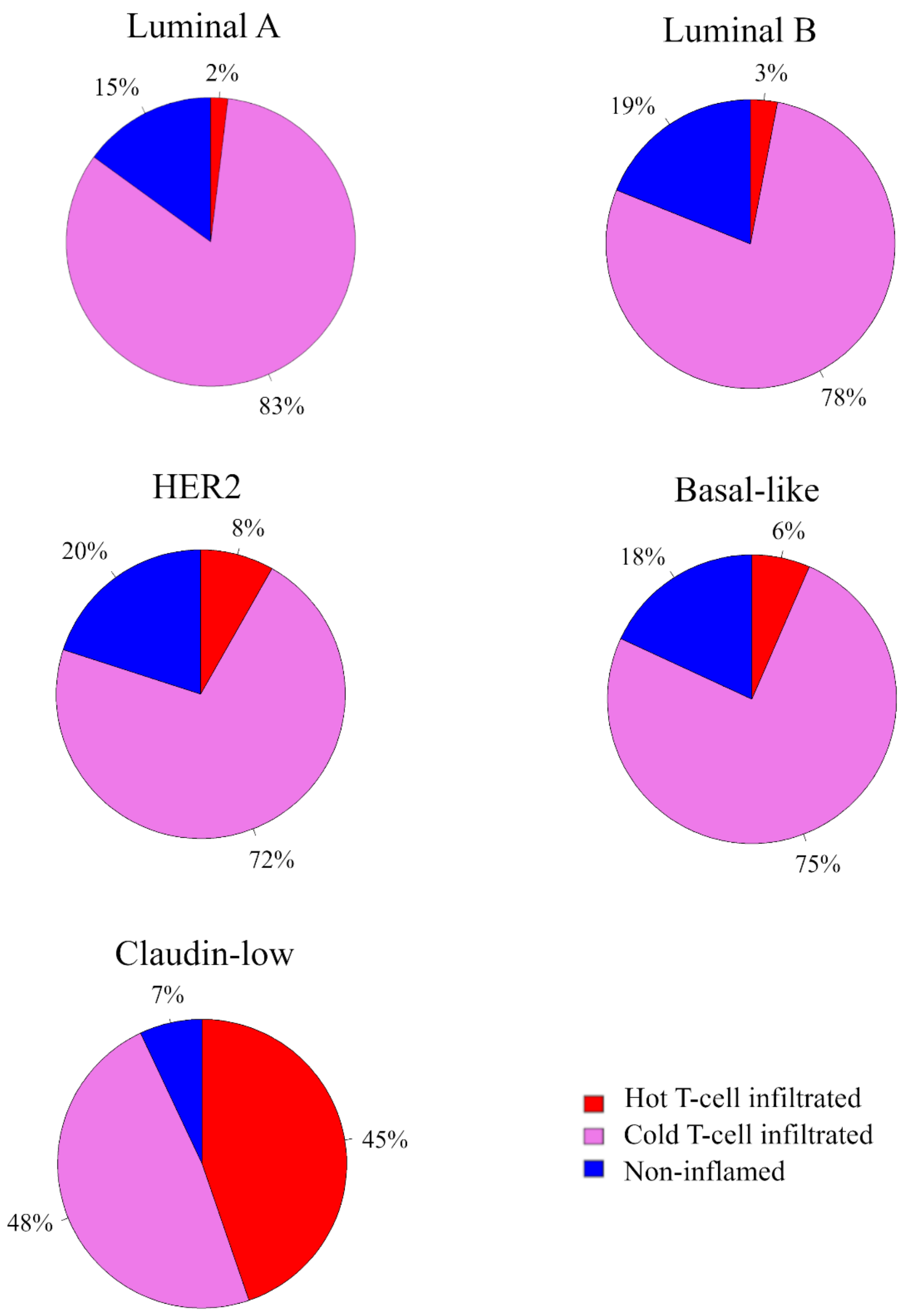

Figure S3. Pie chart of 'Immunofunctional phenotype' in each subtype in METABRIC cohort. Immunofunctional phenotype was categorized as follows: Hot T-cell infiltrated, a group of patients who had high CYT, Cold T-cell infiltrated, a group of patients who had high frequency of CD8+ T cells and low CYT, and Non-inflamed, a group of patients who had low frequency of CD8+ T cells and low CYT. Abbreviations: METABRIC, Molecular Taxonomy of Breast Cancer International Consortium; CYT, immune cytolytic activity.
